# Therapeutic Effect of Decellularized Extracellular Matrix from Fish Skin for Accelerating Skin Regeneration

**DOI:** 10.3390/md22100437

**Published:** 2024-09-26

**Authors:** Seong-Yeong Heo, Tae-Hee Kim, Se-Chang Kim, Gun-Woo Oh, Soo-Jin Heo, Won-Kyo Jung

**Affiliations:** 1 Jeju Bio Research Center, Korea Institute of Ocean Science and Technology (KIOST), Jeju 63349, Republic of Korea; syheo@kiost.ac.kr (S.-Y.H.);; 2 Department of Marine Technology & Convergence Engineering (Marine Biotechnology), University of Science and Technology (UST), Daejeon 34113, Republic of Korea; 3 Research Center for Marine Integrated Bionics Technology, Pukyong National University, Busan 48513, Republic of Korea; 4 Marine Integrated Biomedical Technology Center, The National Key Research Institutes in Universities, Pukyong National University, Busan 48513, Republic of Korea; 5 Major of Biomedical Engineering, Division of Smart Healthcare, College of Information Technology and Convergence and New-Senior Healthcare Innovation Center (BK21 Plus), Pukyong National University, Busan 48513, Republic of Korea; 6 National Marine Biodiversity Institute of Korea (MABIK), Seochun 33662, Republic of Korea

**Keywords:** decellularization, fish skin, olive flounder, skin regeneration

## Abstract

A cellular matrix derived from natural tissue functions as a highly biocompatible and versatile material for wound healing application. It provides a complex and highly organized environment with biological molecules and physical stimuli. Recently, various kinds of tissue/organ decellularized extracellular matrixes (dECMs) from bovine and porcine have been used as biomedical applications to support tissue regeneration but inherit religious restrictions and the risk of disease transmission to humans. Marine fish-derived dECMs are seen as attractive alternatives due to their similarity to mammalian physiology, reduced biological risks, and fewer religious restrictions. The aim of this study was to derive a decellularized matrix from the olive flounder (*Paralichthys olivaceus*) skin and evaluate its suitability as a wound healing application. Olive flounder skin was treated with a series of chemical treatments to remove cellular components. Decellularized fish skin (dFS) was confirmed to be successful in decellularization by evaluating the DNA content (2.84%). The dFS was characterized and evaluated in vivo to assess its biological activities. The mouse wound defect model was used to evaluate the in vivo performance of the dFS compared with that of the decellularized porcine skin (dPS). The resultant dFS was shown to enhance wound healing compared with the no-treatment group and dPS. This study suggests that dFS has potential for skin regeneration application.

## 1. Introduction

The skin is the largest organ in the human body and plays a crucial role in maintaining physiological homeostasis and protecting internal tissues from mechanical insults, foreign chemicals, and environmental pathogens. Therefore, damage to or loss of the skin structure creates a major problem and requires prompt healing [1].

Tissue-engineered skin substitute, as a promising alternative substitute to autograft, has been increasingly used in clinics for wound healing [2,3]. It was designed to biomimic skin construction including its mechanical properties, biological structure, and bioactive molecules in concert to enhance tissue reconstruction [3,4]. However, this method leads to the risk of secondary damage to patients and is also unsuitable for applying to extensive injuries [5]. To address these limitations, many researchers are exploring the application of xenografts for wound healing. Xenografts, as an alternative to autografts, have drawn more attention because it presents not only biological compatibility and functionality but also superior biodegradability [6].

Decellularization technology, a new alternative strategy of tissue engineering, enables the use of native tissue to remove antigen components that have cellular components such as major histocompatibility complex (MHC) and galactose-α-(1,3)-galactose (α-Gal epitope) on the surface of donor cells [7,8]. For that reason, the decellularized extracellular matrix (dECM) has low immunogenicity, high biodegradability, and high biocompatibility. Moreover, it is composed of biological molecules including collagen, glycosaminoglycan (GAG), elastin, and hyaluronic acid and provides suitable structural and mechanical properties [9,10]. Especially, the specific organ-derived dECM can promotes cellular activities including cell proliferation, migration, and differentiation and induces angiogenesis for the provision of oxygen and nutrients because it provides site-specific cellular structure and biological components such as cytokines and chemokines, which control organ functions locally [11,12]. Improvements in decellularization methods have presented new pre-clinical and clinical options for ECM-based functional biomaterials in tissue regeneration.

Fish skin serves numerous functions, such as chemical and physical protection, hormone metabolism, and sensory activity [13]. It has a defense system to protect against pathogens such as bacteria, alga, protozoa, and viruses. This is important because fish are exposed to these pathogens in the aquatic environment, and these same pathogens can colonize an open wound [14]. In particular, skin substitutes from fish skin have attracted attention because there is no risk for viral and prion transmission, whereas mammalian-derived skin grafts require particularly harsh chemical processing to eliminate disease [12]. This harsh processing removes beneficial components such as GAGs, elastin, and collagen. In this regard, fish skin is a promising candidate for human skin regeneration. 

In this study, we evaluated the biocompatibility of the decellularized fish skin and compared wound healing properties between decellularized fish skin (dFS) and decellularized porcine skin (dPS) in wound defect mouse models.

## 2. Results

### 2.1. Decellularization of Fish Skin and Measurement of DNA Content

**Figure 1:**
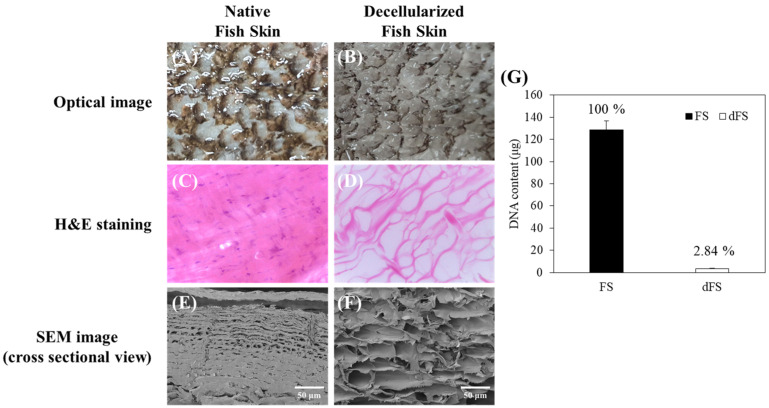
Characterization of (**A**,**C**,**E**) fish skin (FS), and (**B**,**D**,**F**) decellularized fish skin (dFS) and (**G**) DNA content.

After decellularization, a change in the transparency of the fish skin was visually observed. Compared to the native fish skin, cellular cytoplasms and nuclei of cellular components were not detected with electrophoresis and histological evaluation of the dFS. These results confirmed that the decellularization of fish skin by treatment with ionic detergents (0.5% Triton X-100 and 1% SDS) was successful. The H&E staining and SEM images show that the collagen fibers of the dFS are loose compared to the tight and dense collagen fibers in the native fish skin (Figure 1C–F). The surface of the descaled, native fish skin had a smooth and compact layered structure while the dFS had a loose and porous structure with the ECM structure maintained. Moreover, DNA content in the dFS was measured at 2.84% compared with the native fish skin (Figure 1G).

### 2.2. Chemical Composition

**Figure 2:**
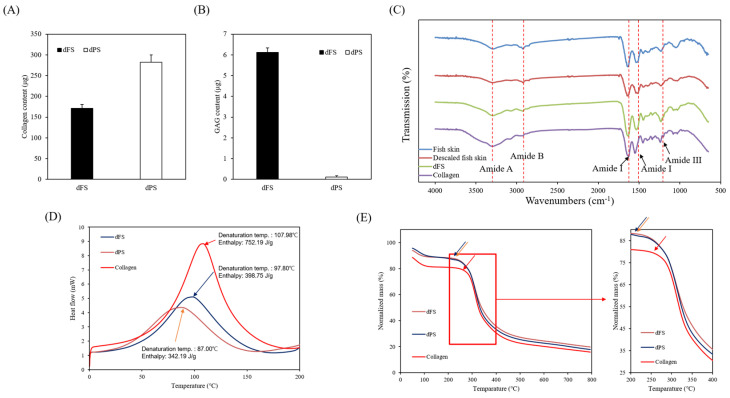
Quantitative analysis of dFS and decellularized porcine skin (dPS), including (**A**) collagen and (**B**) glycosaminoglycans. (**C**) FT-IR spectra of native fish skin, descaled fish skin, dFS, and porcine collagen. Thermodynamic analysis of dFS, dPS, and collagen through (**D**) differential scanning calorimetry (DSC) and (**E**) thermogravimetric analysis (TGA). Data are presented as the mean ± SD (*n* = 3).

The chemical compositions of the fish skin and dFS are shown in Table 1. The dFS was composed of 90.8% protein, 2.7% carbohydrate, 2.9% lipid, 3.6% water, and 0% ash (Table 1). In addition, collagen and glycosaminoglycans in the dFS were measured at 107.95 μg/mg and 6.12 μg/mg, respectively (Figure 2A,B).

### 2.3. FT-IR Analysis

FT-IR was used to analyze the functional components of the ECM of fish skin before and after decellularization, using native fish skin and commercial collagen as a control. Characteristic bands for all samples were observed at 3300 cm^−1^ (N-H stretching), 3070 cm^−1^ (stretching vibrations of N-H), 2924 cm^−1^ (stretching vibrations of N-H), 1651 cm^−1^ (Amide I; C=O stretching), 1543 cm^−1^ (Amide II; N-H deformation), and 1236 cm^−1^ (N-C deformation) (Figure 2C). These FT-IR results suggest that dFS does not undergo chemical modifications and maintains the same components as native fish skin. The dFS also was detected with components similar to commercial collagen.

### 2.4. Thermodynamic Analysis

The thermodynamic properties of dFS and dPS were studied using DSC and TGA. DSC thermograms of the dFS and dPS are shown in Figure 2D and correspond to the heating scan. The results show a transition band at 34.72 °C–144.34 °C, which is due to water loss and the denaturation of protein molecules. In the DSC graph of collagen, this transition band due to water loss and protein denaturation occurs at a higher temperature range. The collagen reached denaturation temperature and an enthalpy change at around 107.98 °C and 752.19 J/g, whereas dFS and dPS showed the same changes at 97.80 °C/398.75 J/g and 87.00 °C/342.19 J/g, respectively. These results indicate that collagen has high thermostability compared to dFS and dPS, which is also supported by the analysis of the TGA curve (Figure 2E). The decomposition temperature was 210 °C for dFS and dPS and 260 °C for collagen.

### 2.5. Tensile Properties

The tensile properties of dFS and native fish skin were evaluated with a UTM. Table 2 shows the mechanical properties of native fish skin and dFS. The results showed that the decellularized tissue became weaker compared to the native fish skin because the collagen fibers have a looser structure.

### 2.6. Wound Closure and Re-Epithelization in Cutaneous Wound

**Figure 3:**
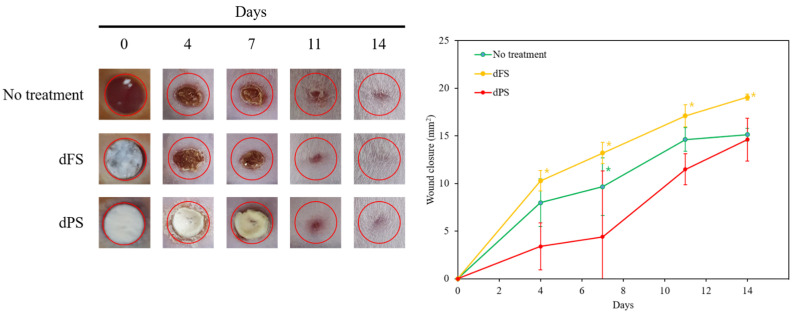
Wound closure images and wound closure on the mouse full-thickness skin wound models for 14 days (0, 4, 7, 11, and 14 days). Data are presented as the mean ± SD (*n* = 6) * *p* < 0.05 indicates a significant difference.

To evaluate the wound healing activity of the dECM samples, 5 mm excisional wounds were made in the dorsal area of mice using a biopsy punch. The dPS and dFS were implanted, and the wounds were observed for 14 days. The mice that received no treatment were used as the control group. The experimental groups were defined as follows: (1) no treatment (control), (2) dFS, and (3) dPS. On Day 4 after transplantation, the initial wound closure was significantly greater in the dFS group compared to that in the no treatment group and the dPS group (Figure 3). 

**Figure 4:**
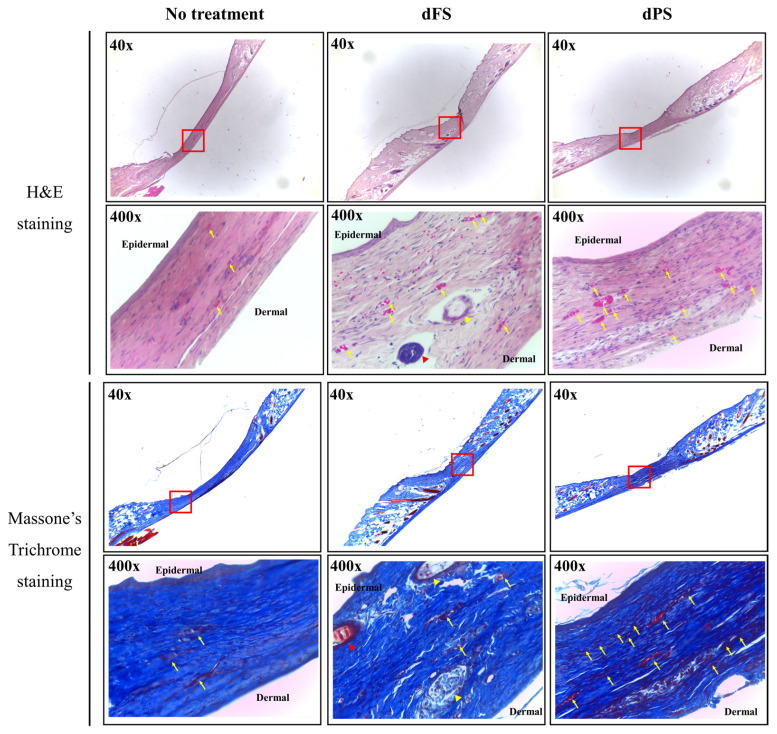
H&E staining and Masson’s trichrome staining of mouse skin 14 days after wounding (yellow arrows indicate blood vessels, yellow arrowheads indicate glands, red arrowheads indicate hair follicles).

**Figure 5:**
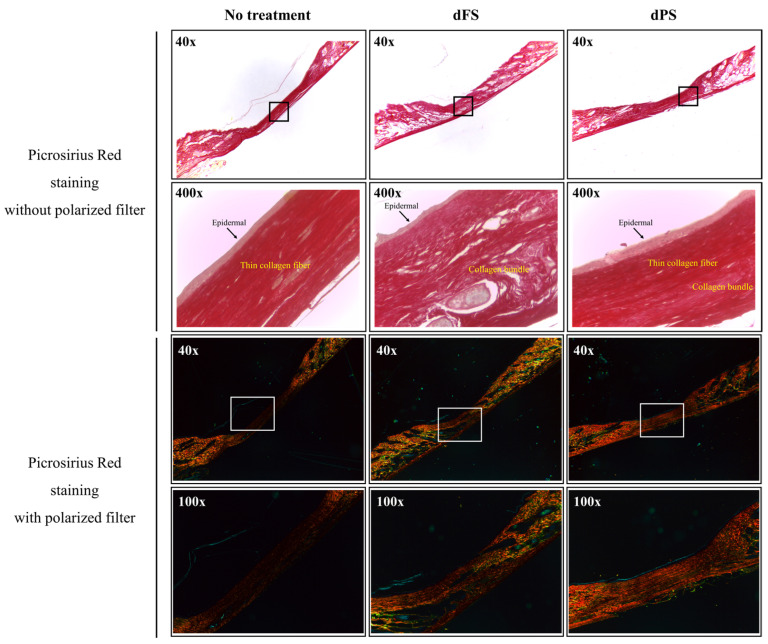
Picrosirius Red staining with or without the polarized filter of mouse skin 14 days after wounding (yellow-orange, type I collagen; green, type III collagen).

**Figure 6:**
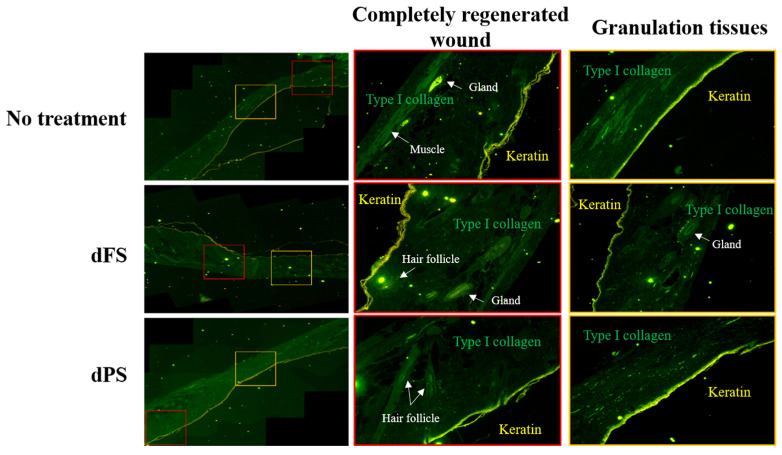
Immunofluorescence staining of mouse skin 14 days after wounding (green, type I collagen; yellow, keratin 10).

On Day 14, the groups treated with decellularized tissues had a significantly smaller wound area compared to that of the no-treatment group. A quantitative analysis based on the photos of the samples indicated that the layered structure of decellularized tissues improves the quality of regenerated skin. Although the wounded tissue of the dFS group was completely regenerated on Day 14 (based on visual observation), scars remained, as indicated by the H&E staining, Masson’s trichome staining, Picrosirius Red staining, and immunofluorescence (Figure 4, Figure 5 and Figure 6).

The dFS group had the highest healing rate, suggesting the potential of dFS for accelerating cutaneous wound healing. The dFS group also had thicker granulation tissue, or regenerated tissue, compared to the other groups. In the dFS group, less granulation tissue was observed in the wound area due to the reconstruction of the skin and skin appendages (gland and hair follicles), whereas for the no-treatment group and dPS group, more granulation tissue remained due to non-healing. Using histological staining, we also found that the wounds treated with dFS achieved relatively quick reconstruction of their ECM by Day 14 (Figure 4 and Figure 5). In Figure 5, many thin collagen fibers are observed in granulation tissue in the dPS group compared to the no-treatment group, whereas the dFS group had thick and matured collagen. As shown in Figure 6, epithelization occurred in all groups, but granulation tissues were still observed in the no-treatment group and dPS group. The dPS group showed large deposits of type I collagen compared to the no-treatment group. These results suggest that dFS promotes the reconstruction of the epidermal and dermal layers.

**Figure 7:**
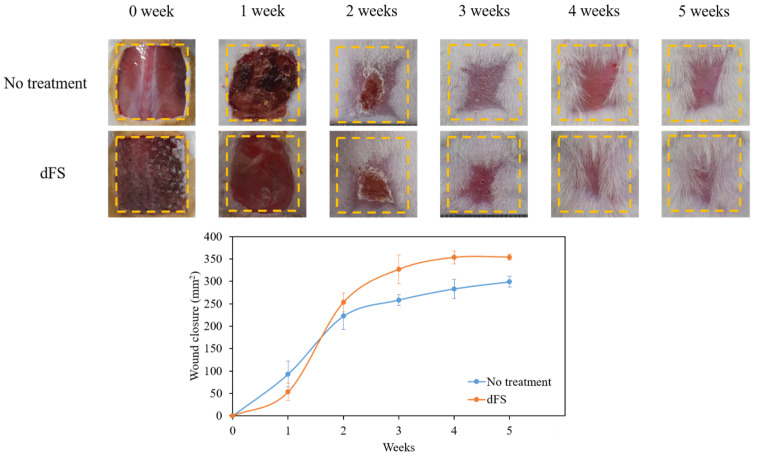
Representative photographs during 5 weeks of wound healing. Data are presented as the mean ± SD (*n* = 6).

**Figure 8:**
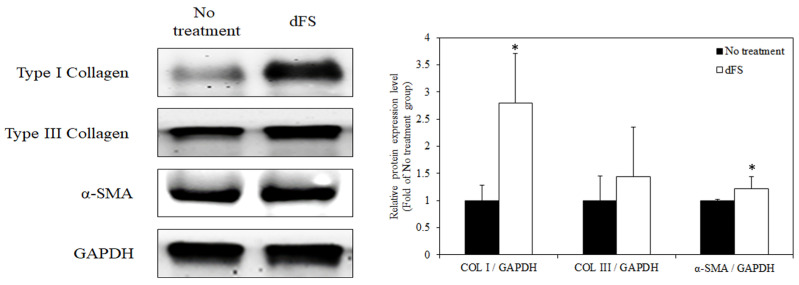
Representative photographs during 5 weeks of wound healing. Data are presented as the mean ± SD (*n* = 6). * *p* < 0.05 indicates a significant difference.

We then initiated a wound healing experiment in which we inflicted standardized, large (20 mm × 20 mm) full-thickness incisional skin wounds on ICR mice to determine whether dFS can be recovered for the critical wound size (Figure 7). The wound lengths were measured every week for 5 weeks. The results showed that the wound area was significantly reduced in the dFS group compared to the no-treatment group after 5 weeks, although the wounds in the control group recovered quickly during the first week. Moreover, the effect of dFS on protein expression levels of skin regenerative phenotype markers was investigated by Western blotting. According to Figure 8, the protein expression levels of type I collagen, type III collagen, and α-smooth muscle actin (α-SMA) in mouse dorsal skin from the dFS group were increased compared to those from the no-treatment group.

## 3. Discussion

Decellularization technology has enabled the use of natural ECM as a biocompatible and functional material for tissue regeneration. This technology provides tissue-specific components including cytokines, chemokines, and growth factors. These facilitate complex tissue formation for performing specific organ functions in the heart, kidneys, and lungs [15,16]. Moreover, a decellularized strategy prevents cell-mediated immunological rejection due to MHC and α-Gal epitope on the surface of donor cells [7,8]. The MHC molecule, located in the cell membrane, distinguishes self vs. non-self-antigen in humans because MHC is equivalent to the human leukocyte antigen (HLA) I [17]. MHC molecules induce both innate and adaptive immune responses that initiate the cytotoxic response of natural killer cells and trigger the activation and amplification of T cell and B cell responses [7]. Other major xenoantigens of the α-Gal epitope have been shown to elicit hyperacute rejection in humans [18]. According to a previous report, all non-primate mammals express numerous glycoproteins and glycolipids through the α-Gal epitope, whereas humans do not express the α-Gal epitope on cell surfaces due to the absence of glycosylation enzyme α-1,3 galactosyltransferase [19]. These studies prove that biomaterials derived from xenogeneic tissue that contains MHC and α-Gal epitope contribute to immune rejection and delayed tissue regeneration.

Recently, many researchers have reported on the use of decellularized allogeneic or xenogeneic ECM from terrestrial animals for biomedical materials for specific tissue regeneration like cardiac [20,21], corneal [22], skin [23], and small intestine [24]. However, medical materials of terrestrial animal origin may pose a safety problem for humans due to potential pathogen contamination and transmissible infective agents such as bacteria residues, viruses, and disease-causing prions [25]. Moreover, the application of terrestrial animal-derived materials, such as collagen and the flu vaccine, may be limited due to constraints in the Muslim, Jewish, and Hindu religions [26]. Therefore, many researchers have shifted focus to marine organism-derived medical materials, which do not pose a risk for disease transmission or religious restrictions. Although many studies have been conducted on biomaterials originating from fish, there are still rarely studies on decellularized fish tissues, and currently, almost all commercially available decellularized products are from terrestrial animals or humans [27,28]. 

Fish-derived tissues are gaining attention as an alternative biomaterial due to their low immunological risk and high availability as a fishery byproduct such as skin, bone, fin, and scale. Heo et al. (2018) reported that teleost bone has similar physiological properties to mammalian bone because it contains similar cellular compositions, including osteocytes and osteoclasts, although it lacks osteons. In addition, osteonectin and calcitonin, which regulate peptides that contribute to osteogenesis, have been found in teleost bone and serve functionally similar roles as in mammalian bone [29]. Interestingly, the composition of fish skin is also comparable to that of human skin [14]. The immune defense system of fish skin is similar to that of humans and includes B lymphocyte-like cells and antigen-specific humoral immunity [14,30]. Therefore, many human disease models have been successfully investigated in teleost fish, especially zebrafish [31,32]. These previous reports indicate significant functional similarities between teleosts and humans. Therefore, the success of using teleost fish in the field of pathology can be extrapolated to the field of tissue engineering, where useful biomaterials could be obtained from teleosts and applied in human tissue regeneration. A previous study showed that decellularized tilapia skin was effective in osteoblast differentiation and calvaria regeneration [27]. In 2014, Remya et al. reported that decellularized fish swim bladder promoted full-thickness skin regeneration in a rat model [28]. Furthermore, Magnusson et al. (2017) showed that acellular fish skin grafts provide anti-bacterial activity and increased ability to induce cell ingrowth [33]. These studies indicate that decellularized fish skin is safe and effective in regenerative biomedical applications, without the disease transmission risk and religious restrictions compared with land animals.

In the present study, we obtained a decellularized ECM matrix from olive flounder skin and evaluated skin regenerative activities through in vivo experiments. After applying a chemical treatment to the fish skin, we determined that the cellular components were successfully eliminated. Figure 1 shows that dFS detected below 3% of the DNA contents (Figure 1G), and did not detect nuclei and cellular components in H&E staining (Figure 1C,D), demonstrating successful decellularization. Figure 1E and 1F show that the collagen bundles of native fish skin have a dense structure, while the collagen structure in dFS was loose due to the effects of ionic detergents, SDS, and Triton X-100 during decellularization. As the collagen form loosened, the mechanical properties and thermostability of dFS significantly reduced compared to those of the native fish skin (Table 2, Figure 2D,E). Figure 2A,B show that dFS had a higher GAG content than dPS. Lohmann et al. (2017) reported that GAG-based materials were attenuated by modulating chemokine in chronic wounds [34]. Moreover, GAG-based matrices increase granulation tissue formation, vascularization, and wound closure [35]. Compared to the dPS group, the dFS group exhibited faster wound regeneration (Figure 3). According to the histological results, the control group had the slowest regeneration because it showed a reconstructed epidermal layer but only a thin dermal layer and widely formed granulation tissues in the wound area (Figure 4). The control group also contained thin collagen fiber, and blood vessels began to form in the wound area. Therefore, the control group may be representative of the proliferation phase in wound healing processes. The dPS group reconstructed many blood vessels to provide oxygen and nutrients and exhibited thin collagen fiber and thick collagen bundles but did not show mature collagen bundles and glands (Figure 5). Therefore, the dPS group represents a remodeling phase in wound healing processes. The dFS group contained blood vessels and gland and hair follicles. It also had a thick dermal layer and thick collagen bundles. Fewer blood vessels were observed in the dFS group than in the dPS group because blood vessels regress in the skin tissue after remodeling has been completed [36]. Furthermore, outcomes of an in vivo study showed higher expression levels of type I collagen, type III collagen, and α-SMA, representative markers related to skin regeneration in dorsal granulation tissues from mice with dFS (Figure 8). These results suggest that dFS is a promising material to promote skin regeneration in biomedical applications.

## 4. Materials and Methods

### 4.1. Materials

Olive flounder (*P. olivaceus*) skins were obtained from EUNHA Marine Co. Ltd. (Busan, Republic of Korea). The 3-(4,5-dimethylthiazol-2-yl)-2,5-diphenyltetrazolium bromide) (MTT) were purchased from Sigma-Aldrich, St. Louis, MO, USA. Dulbecco’s minimum Eagle’s medium (DMEM), fetal bovine serum (FBS), trypsin (250 U/mg), penicillin/streptomycin, and other materials used in the cell culture experiment were purchased from GIBCO™, Invitrogen Corporation, Carlsbad, CA, USA. α-SMA (sc-53142), GAPDH (sc-66163), and keratin 10 (sc-53252) were purchased from Santa Cruz Biotechnology (Santa Cruz, CA, USA), and type I collagen (ab316222) and type III collagen (ab184993) were purchased from Abcam (Cambridge, UK). All other chemicals and solvents were analytical-grade, and the water used in the experiments was deionized.

### 4.2. Preparation of Decellulairzed Extracellular Matrix from Olive Flounder

The decellularization of the fish skin was performed according to the previous report with slight modification [27]. Freshly harvested skin from olive flounder (*P. olivaceus*) was cleaned, de-scaled, and rinsed in PBS for 2 h. Fish skin was immersed in 0.5% Triton X-100, followed by incubation for 9 h at 37 °C. After incubation, the debris were physically removed from the fish skin and rinsed in PBS for 2 h. The fish skin was treated with 1% sodium dodecyl sulfate (SDS) in PBS solution for 3 h. The treated fish skin was then rinsed in PBS for 2 h to remove the detergent. Next, the fish skin was sterilized with 0.1% peracetic acid in PBS and was washed several times with PBS. The obtained dFS was lyophilized and stored at −80 °C in a freezer.

### 4.3. Approximate Chemical Composition

The approximate chemical composition was determined using the Association of Official Analytical Chemists (AOAC) method [37]. The crude protein content was determined by the Kjeldahl method, crude carbohydrate quantity was determined by phenol-sulfuric acid reaction (absorbance at 480 nm, using glucose as the calibration standard), crude lipid content was measured using the Soxhlet method, and crude ash was prepared at 550 °C in a dry-type furnace for 3 h [38,39].

### 4.4. DNA Content and Histological Assessment

For DNA assessment, the DNA was extracted as described by Keane et al. (2012) but with slight modifications [40]. Native fish skin and decellularized fish skin (dFS) were digested with proteinase K digestion buffer (100 mM NaCl, 10 mM Tris-HCl pH 8.0, 25 mM EDTA pH 8.0, 0.5% SDS, 0.1 mg/mL proteinase K) at 50 °C for 48 h. The digested DNA was extracted twice using phenol/chloroform/isoamyl alcohol (25:24:1). The aqueous layers were removed, and ethanol precipitated at −20 °C for at least 8 h to isolate any DNA. The DNA was then centrifuged at 10,000× *g* for 10 min and resuspended in 1 mL of TE buffer (10 mM Tris pH 8.0, 1 mM EDTA). DNA content was measured at 260 nm using a microplate spectrophotometer (PowerWave XS2, BioTek Instruments, Inc., Winooski, VT, USA) according to Lambert–Beer law.

For histological evaluation, both the native and decellularized tissues were fixed in 4% formalin. Then, the tissues were processed routinely for 14 h through graded ethyl alcohol, xylene, and paraffin using a Leica TP1020 tissue processor (Leica Biosystems, Nussloch, Germany). Next, paraffin embedding was performed using a Leica EG1160 Paraffin Embedding Center (Leica Biosystems, Nussloch, Germany). The samples were then sectioned using a Leica RM2245 rotary microtome (Leica Biosystems, Nussloch, Germany), stained with H&E and immunofluorescence, and observed under a microscope. 

### 4.5. Glycosaminoglycans (GAGs) Content Analysis

The total sulfated GAGs content of the native fish skin tissue and dFS tissue was quantified using a Blyscan sulfated glycosaminoglycan assay kit (Biocolor, Carrickgergus, UK) according to the manufacturer’s instructions. First, 1 mg of freeze-dried samples was digested with 1 mL of papain extraction buffer at 65 °C for 1 day. After this, each tube contained 100 μL of test samples, standards, or a blank control. Then, 1 mL of Blyscan dye reagent was added and mixed for 30 min using a mechanical shaker, followed by centrifugation at 12,000 rpm for 10 min. The supernatant was carefully removed, and 1 mL of dissociation reagent was added to the tubes and left to dissolve for 10 min before measurement. The absorbance of the reagent blanks, GAGs standards, and test samples was measured at 656 nm using a microplate spectrophotometer. Absolute values were determined from a standard graph that was prepared using the supplied GAGs standards in the range of 1–5 μg per 0.1 mL.

### 4.6. Collagen Content Analaysis

A Sircol soluble collagen assay kit (Biocolor, Carrickgergus, UK) was used to isolate and quantify the collagen content of the samples according to the manufacturer’s instructions. First, 1 mg of freeze-dried sample was hydrolyzed with 1 mL of 0.1 mg/mL pepsin solution in 0.5 M acetic acid at 4 °C overnight. After neutralization, the collagen was isolated using the supplied isolation and concentration reagent. Then, 100 μL of the sample was added to 1 mL of colorimetric reagent and agitated for 30 min, followed by centrifugation at 12,000 rpm for 10 min. The dye was released from the pellet using the supplied alkali reagent, and the absorbance at 555 nm was measured using a microplate reader. Absolute values were obtained from a standard graph that was made using the type I collagen standard supplied with the kit in the range of 5–100 μg per 0.3 mL.

### 4.7. Analysis of dECM Morphology

The morphology of the dECM samples was visualized using a field emission scanning electron microscope (FE-SEM, Hitachi S-2700, Tokyo, Japan) at 2.0 kV after the samples were sputter-coated with silver using Emi-Tech K500X (Emitech, Ruelle-sur-Touvre, France).

### 4.8. FT-IR

FT-IR spectroscopy (FT-4100, JASCO, Tokyo, Japan) was used to measure the relative concentration of the dECM samples. The spectra were measured with a resolution of 4 cm^−1^ in the frequency range of 4000–650 cm^−1^ at room temperature.

### 4.9. Analysis of Mechanical Properties

The tensile properties of the samples were measured using a universal testing machine (UTM, Top-tech 2000, Chemilab, Republic of Korea). The samples were mounted and subjected to a crosshead speed of 0.5 mm/min at room temperature until breakdown was observed. Each sample was tested in triplicate, and the averages for max tensile strength, maximum stress, and Young’s modulus were calculated.

### 4.10. Thermal Analysis

Calorimetric measurements were conducted using differential scanning calorimetry (DSC, Perkin Elmer, Diamond, Waltham, MA, USA) under nitrogen flowing at a rate of 10 mL/min. The specimens were pressed in sealed aluminum pans. A heating cycle was performed to reach the glass transition temperature (T_g_) and melting temperature (T_m_). During this cycle, the samples were heated from 0 °C to 250 °C at a rate of 10 °C/min. The samples were then cooled down by nitrogen at an exponentially decreasing rate.

The thermogravimetric analysis (TGA) scans of the samples were carried out using Perkin Elmer Pyris 1 instrument (Perkin-Elmer Analytical Instruments, Norwalk, CT, USA) to detect the mass variation. TGA experiments were performed at a heating rate of 10 °C/min in a temperature range from 50 °C to 800 °C under inert atmosphere (10 cc/min of nitrogen).

### 4.11. In Vivo Cutaneous Wound Healing on Animal Model After dFS Implantation

All animal experiments were performed in accordance with the protocols approved by the Institutional Animal Care and Use Committee of Pukyong National University (PKNUIACUC-2019-19). The 6-week-old, male, Institute of Cancer Research (ICR) mice were purchased from Orient (Seongnam, Republic of Korea). The mice were randomly divided into the following three groups (*n*  =  6 per group): no treatment (control), dFS, and dPS. The mice were anesthetized with 20% isoflurane in isopropanol for 20 min, and a full-thickness, excisional dorsal wound was physically created using a 5 mm biopsy punch and sterile surgical scissors [41,42]. The wound site was treated with dECM samples on Day 0. Immediately following this structure implantation, the wounds were covered with a dressing film (TegadermTM Film, Saint Paul, MN, USA) to protect them from dryness and damage from self-grooming. On days 0, 4, 7, 11, and 14 post-wounding, a digital camera was used to take photographs of the wound sites. The wound areas in each group were measured using Image J software Version 1.54 (Wayne Rasband, NIH, Bethesda, MD, USA) and then compared with the non-treatment wound at each time point.

### 4.12. Histological Analysis In Vivo

The samples were fixed with 10% neutral buffered formalin. The fixed samples were washed overnight with running tap water and dehydrated in ascending concentrations of alcohol. They were then cleared in xylene and infiltrated in paraffin using a tissue processor (TP 1020, Leica Biosystems, Wetzlar, Germany). Next, the samples were embedded in paraffin and sectioned with a thickness of 5 μm. The paraffin sections were deparaffinized by keeping the sections in a dry oven at 40 °C overnight and were cleared by dipping in xylene for 30 min. Then, the samples were rehydrated in decreasing concentrations of alcohol (100% and 95%) for 3 min each and washed in distilled water (DW) for 5 min.

The paraffin sections were stained using H&E (MHS16 hematoxylin, Sigma-Aldrich, USA; Eosin Y; 17025-1210, Junsei, Tokyo, Japan). Then, the samples were dehydrated again by gradual immersion in alcohol (95% followed by 100%), dipped in xylene, and mounted in mounting solution.

For Sirius red staining, the paraffin sections were stained with 0.1% Sirius red solution in saturated aqueous picric acid for 90 min. Then, the samples were washed with 1% acetic acid, dehydrated by gradual immersion in alcohol (95% followed by 100%), dipped in xylene, and mounted in mounting solution.

Paraffin sections were also stained with Masson’s trichrome stain (Masson’s Trichrome stain kit; KTMTR, American MasterTech, Lodi, CA, USA) to quantify wound regeneration. The samples were incubated in a Weigert’s A&B hematoxylin bath for 10 min, washed in DW, incubated in a Biebrich scarlet solution for 5 min, and rinsed with DW. The paraffin sections were incubated with phosphotungstic/phosphomolydbic acid for 10 min and immediately transferred to aniline blue solution for 5 min. Then, the samples were rinsed with DW for 5 min, 1% acetic acid for 1 min, and DW again. Finally, the paraffin sections were mounted in a mounting solution.

For Sirius red staining, the paraffin sections were stained with 0.1% Sirius red solution in saturated aqueous picric acid for 90 min. Then, the samples were washed with 1% acetic acid, dehydrated by gradual immersion in alcohol (95% followed by 100%), dipped in xylene, and mounted in mounting solution. Then, specimens were detected using a fluorescence microscope with polarized filter. Under polarized light, type I collagen fibers and type III collagen fibers were detected red or orange-yellow and green, respectively.

For immunofluorescence imaging, paraffin sections were incubated in 10 mM sodium citrate buffer (pH 6.0) for 30 min and rinsed in DW three times. The samples were placed in 3% H_2_O_2_ solution, washed with DW, and incubated in TBST buffer for 5 min. Then, the paraffin sections were incubated in blocking buffer at room temperature for 1 h. Next, the paraffin sections were incubated overnight with the primary antibody anti-keratin 10 and anti-type I collagen (Abcam, Cambridge, UK) at 4 °C. The samples were washed in TBST three times and incubated for 2 h at room temperature with the secondary antibody Alexa 633 and the Alexa 546 conjugated, anti-rabbit, and anti-mouse antibodies (Invitrogen, Carlsbad, CA, USA). Nuclear counterstaining was performed using Hoechst stain. Lastly, immunofluorescence specimens were detected using a fluorescence microscope (Axio Observer A1, Zeiss, Jena, Germany).

### 4.13. Statistical Analysis

All quantitative data are presented as means ± standard deviation and represent at least three individual experiments conducted using fresh reagents. Statistical comparisons of the mean values were performed using analysis of variance (ANOVA) followed by Duncan’s multiple range test using IBM SPSS software Statistics 30.0.0. Differences in mean values were considered statistically significant at * *p* < 0.05, ** *p* < 0.01.

## 5. Conclusions

In conclusion, we developed decellularized fish skin by treatment of ionic detergent and evaluated its biochemical and physical characteristics. The skin of olive flounder was decellularized to produce skin-regenerative biomaterial, and its biochemical and physical characteristics were analyzed. Fish dECM was evaluated in vivo to assess its biological compatibility and suitability as a tissue-regenerative material. The results indicated that fish dECM possesses biocompatibility with host tissues and promotes wound closure by increasing the expression of regenerative skin phenotype markers, including type I collagen, type III collagen, and α-SMA. This study demonstrates the high potential of fish-derived biomaterials as an economical and abundant option for medical applications without the risk of immune rejection and disease transmission.

## Figures and Tables

**Table 1:** Chemical composition of native fish skin and decellularized fish skin.

	Native Fish Skin (g/100 g)	Decellularized Fish Skin (g/100 g)
Protein	90.9	90.8
Carbohydrate	0.4	2.7
Lipid	3.5	2.9
Moisture	4.0	3.6
Ash	1.2	0

**Table 2:** Mechanical properties of native fish skin and decellularized fish skin.

	Maximum Stress (MPa)	Tensile Strength (MPa)	Young’s Modulus (MPa)
Native fish skin	32.19 ± 2.11	32.30 ± 0.51	240.24 ± 16.20
Decellularized fish skin	6.07 ± 1.24	6.80 ± 1.15	92.89 ± 11.17

## Data Availability

The data presented in this study are available on request from the corresponding author.
